# Phylogeny of *Catabacter hongkongensis* Strains Responsible for Bacteremia Is Not Associated with Clinical Outcomes or Therapeutic Efficacy

**DOI:** 10.3390/diseases9020024

**Published:** 2021-03-25

**Authors:** Matthieu Cabrol, Blandine Rammaert, Chloé Plouzeau, Christophe Burucoa, Maxime Pichon

**Affiliations:** 1 Département des Agents Infectieux, Laboratoire de Bactériologie, CHU de Poitiers, 86021 Poitiers, France; m.cabrol@medilab-group.eu (M.C.); chloe.plouzeau-jayle@chu-poitiers.fr (C.P.); christophe.burucoa@chu-poitiers.fr (C.B.); 2 Service de Maladies Infectieuses et Tropicales, CHU de Poitiers, 86021 Poitiers, France; blandine.rammaert@chu-poitiers.fr

**Keywords:** *Catabacter hongkongensis*, sepsis, phylogeny, 16S rDNA sequencing

## Abstract

*Catabacter hongkongensis* is a Gram-positive rod, isolated in 2007 in blood culture. Fewer than 15 infections have been reported. Herein, we present a lethal case of bacteremia due to *C. hongkongensis* identified through phylogenetic analyses. A woman was found unconscious in a context of chronic diarrhea. An abdominal abscess with a hydroaeric level was discovered, associated with sigmoid adenocarcinoma and peritoneal carcinomatosis. Despite hospitalization in an ICU and the adaptation of antibiotic therapy, the patient died. Blood cultures were positive in the final stage of the disease (>60 h). Identification of *C. hongkongensis* was performed using 16S rDNA sequencing. Phylogenetic analyses did not enable classification of these strains according to clinical outcome or the antibiotic sensitivity to treatment. In this case, bacteria were difficult to isolate and MALDI-TOF remained non-contributive. As strains are resistant to probabilistic treatments, addition of metronidazole or vancomycin could optimize clinical management, highlighting the benefit of rapid molecular identification by sequencing.

## 1. Introduction

*Catabacter hongkongensis* (*C. hongkongensis*) is a strict anaerobic and non-sporuled Gram-positive rod [1]. Since its initial isolation in 2007 in the blood cultures of Hong Kong (six samples) and Canadian patients, no more than 15 infections have been identified in humans [2]. Even though its genome has been detected in environmental or animal samples, only three cases have been reported in Europe (France, Sweden and Italy) [3,4,5,6]. The relatively low number of cases could suggest that this bacterium is underdiagnosed in clinical observation, mostly due to its cultural traits and long delay of culture (median time of detection in blood culture of 72 h, and a minimum time to a positive result in blood of 48 h). Considering the low prevalence of these infections and their association with underlying diseases, they are potentially very severe, leading to septic shock in the context of gastrointestinal diseases (intestinal occlusion, appendicitis, intestinal perforation and cholecystitis) [7].

Herein, we present a lethal case of bacteremia due to *C. hongkongensis*. This manuscript is the second to describe such a case in France, including, for the first time, phylogenetic analyses supported by clinical comparison to literature.

## 2. Experimental Section

An 80-year-old woman, self-supporting but with a history of alcoholic cirrhosis with cholestatic hepatitis, was found unconscious at home. She was addressed to the emergency room of the University Hospital of Poitiers, France. The clinical history of the patient included diarrhea (without blood or glair) for 15 days, associated with anorexia, diffuse edemas and global alteration. Clinical examination revealed high fever (39 °C), major asthenia with cachexia (low albuminemia 21.5 g/L), leg edemas and hypokalemia (2.7 mM) without cardiac rhythm alteration on the ECG. Given her clinical presentation, the patient was hospitalized and, after biochemical and microbiological sampling, a preemptive treatment by third-generation cephalosporin (intravenous ceftriaxone) was introduced, associated with potassium supplementation and furosemide. On day 2 blood culture was positive for *Staphylococcus aureus* in a blood culture sampled on day 1 (time to positive result: 13.5 h) and confirmed on another hemoculture sampled on day 4 (time to positive result: 21.6 h). Antibiotics were modified to introduce first-generation cephalosporin (cefazoline) and aminoglycoside (gentamicin) for 14 days. Antibiotic susceptibility testing of this *S. aureus*, using disk diffusion method, showed susceptibility to all antibiotics except macrolides (MIC > 4.0µg/mL). On the same day, radiological examination (thoraco-abdominopelvic tomodensitometry) was performed and revealed an abdominal abscess with a hydroaeric level (77 × 54 × 36 mm^3^), fistulized to a sigmoid tumefaction (68 × 67 × 60 mm^3^). Given these radiological images, adenocarcinoma associated with peritoneal carcinomatosis and effusion lamella in the Douglas cul-de-sac was diagnosed.

Due to the persistence of the fever, anaerobic and aerobic bacterial vials were sampled daily (BACT/ALERT^®^ 3D, bioMérieux, Marcy-l’étoile, France). After 67 and 74 h of culture at 35 °C, two anaerobic bottles were positive. On direct examination after gram staining, Gram-positive coccobacillus bacteria were identified. They were sub-cultured on Columbia with 5% sheep blood (bioMérieux), obtaining a fine culture in 48 h. Even though bottles were re-seeded with a high sample volume, no identification could be performed using MALDI-TOF MS (VITEK^®^ MS, bioMérieux), thereby justifying the application of sequencing procedures (Sanger sequencing of the DNA coding the 16S rRNA V1-V3 regions on 3500 Dx Genetic Analyzer, Thermo Fisher Waltham, MA, USA) to identify *C. hongkongensis*. Antibiotic sensitivity testing (AST) (testing for sensitivity to amoxicillin, tazocillin and piperacillin, imipenem, clindamycin, metronidazole, moxifloxacin, vancomycin and ceftazidime) was carried out on a diffusion mode according to CA-SFM-EUCAST recommendations (Société Francaise de Microbiologie; March 2017). The bacteria strain was tested for amoxicillin (Minimum Inhibitory Concentrations, MIC = 1 mg/L, S), tazocillin and piperacillin (MIC = 16 mg/L, I), imipenem (MIC = 0.094 mg/L, S), clindamycin (MIC < 4 mg/L, S), metronidazole (MIC < 4 mg/L, S), moxifloxacin (MIC < 1 mg/L, S) and vancomycin (MIC < 2 mg/L, S), and was found to be resistant to ceftazidime (MIC > 8 mg/L, R). One month after her hospitalization, following adaptation of antibiotic therapy with piperacillin–tazobactam and vancomycin according to an anesthesiologist consensus conference (in front of severe digestive infection), the patient died.

## 3. Results

**Figure 1:**
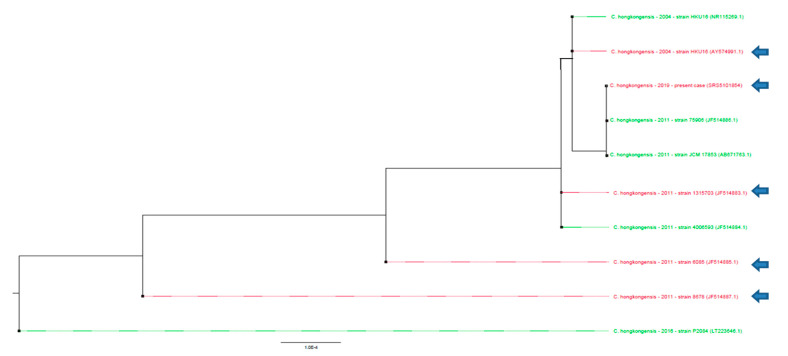
Phylogenetic analysis of *Catabacter hongkongensis* identified in blood cultures. Phylogenetic tree drawn up by ML-Method on 631 bases, on available sequences. An arrow indicates strains of *Catabacter hongkongensis* responsible for lethal infections. No significant association of these sequences could be obtained in terms of severity (*p* > 0.05) as no clustering could be statistically observed. No significant clustering could be observed regarding the antibiotic sensitivity testing (AST) profile using 16S rDNA sequencing phylogenetic analysis (*p* > 0.05).

The sequence produced during Sanger sequencing was retrospectively analyzed using SeaView Version 4.7 to compare the 16S rRNA V1–V3 sequence of the bacteria strain with the sequences published in the literature [8]. The maximum likelihood phylogenetic trees (1000 bootstraps) of these partial 16S DNA sequences were not sufficient to classify these strains according to either the clinical outcome, the AST or the efficacy of the treatment (Figure 1.) (*p* > 0.05) [9]. The sequence has been deposited onto GenBank (accession number SRS5101854).

## 4. Discussion

Herein, we presented a rare case (to our knowledge, fewer than fifteen cases have been reported in the literature) of bacteremia by *C. hongkongensis* that was associated with the patient’s death during the clinical management. These opportunistic environmental bacteria have been associated with severe bacteremia (lethality rate: 40%, seven lethal cases out of 16 published cases), in the context of neoplasia (six of 16 published cases; 37.5%) (Table 1). The species, *Catabacter* sp., has previously been detected in the gastrointestinal microbiota, although no precise details about *Catabacter hongkongensis* were given [10]. It was observed that clinical presentation of these infections mostly implicated peritoneal organs and was associated with digestive translocation in the context of inflammation (Table 1). In all described cases, authors stated that these bacteria could be difficult to isolate, with a median time of detection in blood culture of 72 h (from 2 to 5 days), similar to potential sample contaminants, and difficult to identify [1]. However, due to their absence from the skin microbiota (never described in microbiota studies on skin biopsies, even using culture-independent methods such as ultra-deep sequencing), identification in blood culture necessitates the need for rapid identification and antibiotic susceptibility testing according to recommendations [11]. It is important to note that in the present case, the bacteria were identified twice and the blood culture time to positive results was shorter (below 24 h for both positive vials) than in the literature. This delay could reinforce the clinical responsibility of this bacteremia in the negative outcome of the patient, reflecting a higher bacterial load in the blood sample. Nevertheless, the role of this bacteria in the patient’s death cannot be completely assumed, as the age and multiple comorbidities of the patient must be considered. Independent of the quality and easy-to-obtain characteristics of the subculture, MALDI-TOF has been unable to identify *C. hongkongensis*, of which the absence in MALDI-TOF libraries could be responsible for an analytical bias and could constitute an explanation for the low representation of this pathogen in human pathologies in general, especially in bacteremia. In this context, bacterial 16S rDNA sequencing could be of major interest as a means of identifying these bacteria in routine management (more than 16% dissimilarity with phylogenetically close bacteria, such as *Lactobacillus*, *Eubacterium* or *Clostridium*) [1]. While application of such a method in clinical microbiology practice could be difficult to organize, easy-to-use software could help in analyzing sequences that could be obtained in a 6 or 8 h period of time [8]. This approach could be considered as key for the identification of such bacteria and would allow the efficient adaptation of the clinical management of the patient However, phylogenetic analyses produced by this Sanger sequencing approach cannot be used to predict the efficacy of anti-microbial therapies in bacteria with high mutation rates such as *Enterobacteriaceae*. Finally, even though phylogenetic analysis through sequencing is of major interest as a means of identifying the pathogen, and could be used for some bacterial or viral strains to predict severity of the disease, it was of no interest in the present case [12]. Nevertheless, in these situations of clinical importance, and due to the democratization of rapid Next-generation Sequencing (NGS) technologies, it could be discussed to analyze such bacteremia using whole-genome sequencing, allowing the identification of resistance-associated and virulence-associated genes of the bacterial strains.

Clinical management of the described patient was justified by the anesthesiologists’ consensus conference [14]. In ICU, anesthesiologists who managed the patient have adhered to this consensus, which stated the recommendation of an association of amoxicillin/clavulanate or cephalosporin/metronidazole for five to seven days, with rapid adaptation as soon as possible (modified to quinolone/gentamicin/metronidazole when allergy is suspected or modified to piperacillin/tazobactam/gentamicin in cases of severe disease) [14]. As summarized in Table 1, while more than one third of the reported strains could be resistant to these (penicillin- and cephalosporin-resistant) probabilistic treatments, the addition of metronidazole of vancomycin may enable effective treatment. It could be of interest to ascribe the therapeutic failure in the first days of the treatment, as described, to the high lethality of these infections (6/15; 40%).

## 5. Conclusions

This case of lethal infection by *C. hongkongensis* highlights the importance of adapted preemptive antibiotic treatment in cases of severe sepsis. It also underlines the benefit of rapid molecular identification by sequencing (next-generation sequencing or conventional methods) as a complement to routine diagnosis methods such as MALDI-TOF or biochemical identification.

## Figures and Tables

**Table 1:** Clinical presentations of *Catabacter hongkongensis* bacteremia. TTP: time to positive results; CEF: cefotaxime; MET: metronidazole; MIC: minimum inhibitory concentration; ND: non-determined; -: sequences were not reported; PEN: penicillin; R: resistant; S: susceptible; VAN: vancomycin, S*: absence of the gene associated with antibiotic resistance. All the described cases were associated with gastrointestinal diseases. These fifteen cases highlight the severity of infection by *Catabacter hongkongensis*. This severity is independent of the undertaken clinical management. The antibiotic susceptibility testing of the isolated strains demonstrates: i/ the inconsistent effectiveness of beta-lactamines (including cephalosporins) and ii/ the constant efficacy of vancomycin and imidazole.

Age (Years)	Sex	Place of Identification	Main Diagnosis	TTP (days)	Antibiotic Treatment	Patient’s Issue	Antibiotic Susceptibility Testing (SIR Status, MIC in µg/mL)					GenBank Accession Number (Sequence)	Reference
							VAN	PEN	CEF	MET	AST Methods		
48	M	China	Bowel obstruction	3	Cefuroxime Metronidazole	Survival	S (2)	S (0.75)	R (>32)	S (<0.016)	E-test	-	[1]
39	M	China	Appendiceal perforation	3	Cefuroxime Metronidazole Surgery	Survival	S (2)	S (0.5)	R (>32)	S (<0.016)	E-test	-	[1]
74	M	Canada	Fever associated with a biliary stent in a context of plasmocytosis	NR	Ciprofloxacin	Survival	S (2)	R (4)	R (>32)	S (<0.016)	E-test	-	[1]
66	F	Canada	Infection on lung cancer	5	Cefuroxime Ciprofloxacin	Death	S (2)	R (4)	R (>32)	S (<0.016)	E-test	AY574991	[1]
52	M	France	Septic shock on intestinal perforation	3	Amoxicillin Clavulanate Gentamicin Surgery	Death	S (<0.016)	R (2)	R (>32)	S (<0.016)	E-test Disk Diffusion	-	[4]
47	M	New Zealand	Appendiceal perforation with perineal abscess	4	Amoxicillin Clavulanate Cefuroxime Metronidazole Surgery	Survival	NR	R (4)	S (ND)	S (ND)	E-test	AB671763.1	[2]
91	F	China	Sepsis with hepatic abscess on colic adenocarcinoma	3	Ticarcillin Clavulanate Gentamicin	Death	S (1)	S (<0.016)	R (>32)	S (<0.016)	E-test	JF514883	[7]
21	M	China	Gangrene on appendiceal perforation	3	Cefuroxime Metronidazole	Survival	S (1)	S (<0.016)	R (>32)	S (<0.016)	E-test	JF514884	[7]
81	F	China	Metastatic colonic adenocarcinoma	3	Amoxicillin Clavulanate Tazobactam Piperacillin	Death	S (1)	S (<0.016)	R (>32)	S (<0.016)	E-test	JF514885	[7]
76	M	China	Acute cholecystitis	3	Cefuroxime Metronidazole	Survival	S (1)	S (<0.016)	R (>32)	S (<0.016)	E-test	JF514886	[7]
81	F	China	Metastatic colonic adenocarcinoma	3	Cefuroxime Metronidazole	Death	S (1)	S (<0.016)	R (>32)	S (<0.016)	E-test	JF514887	[7]
55	M	Italy	Septic shock	4	Vancomycin Meropenem	Survival	S (1)	NR	NR	S (<0.016)	E-test	-	[5]
77	M	Korea	Acute cholecystitis	3	Cefodizime Metronidazole Surgery	Survival	S (0.75)	R (>32)	R (>32)	S (ND)	E-test Disk Diffusion	LT223646	[13]
83	M	Sweden	Isolated fever	3	None	Survival	S*	S*	S*	S*	Genotypic Determination	-	[6]
80	F	France	Febrile diarrhea on sigmoid adenocarcinoma	2–3	Cefazolin Gentamicin	Death	S (<2)	S (1)	R (>32)	S (<4)	E-test Disk Diffusion	SRS5101854	Present Case

## Data Availability

Newly produced sequences has been deposited onto GenBank (accession number SRS5101854).
